# The Improving Global Health fellowship: a qualitative analysis of innovative leadership development for NHS healthcare professionals

**DOI:** 10.1186/s12992-018-0384-3

**Published:** 2018-07-17

**Authors:** Alexandra Monkhouse, Leanne Sadler, Andrew Boyd, Fleur Kitsell

**Affiliations:** 1 A Monkhouse Ltd, London, UK; 2 Cardiff and Vale University Health Board, London, UK; 3 Clapham Park Group Practice, London, UK; 4 Thames Valley and Wessex Leadership Academy, London, UK

**Keywords:** Leadership development, Career development, Global health, Personal development, Qualitative research, Overseas partnerships

## Abstract

**Background:**

The importance of leadership development in the early stages of careers in the NHS has been highlighted in recent years and many programmes have been implemented which seek to develop leadership skills in healthcare professionals. The Improving Global Health (IGH) Fellowship scheme is one such programme, it provides a unique leadership development opportunity through an overseas placement with a focus on quality improvement work. This evaluation examines the impact of completing an IGH Fellowship on the career and leadership development of participants, who are referred to as Fellows.

**Methods:**

Fellows who had returned from overseas placement between August 2008 and February 2015 were invited to complete an anonymised online questionnaire, which collected information on: demographic details, motivations for applying to the programme, leadership development and the impact of the IGH Fellowship on their career. Fifteen semi-structured interviews were conducted to further explore the impact of the programme on Fellows’ leadership development and career progression. Interview transcripts were manually coded and underwent thematic content analysis.

**Results:**

The questionnaire had a 67% (74/111) response rate. The number of fellows who self-identified as a leader more than doubled on completion of the IGH Fellowship (24/74 pre-fellowship versus 58/74 post-fellowship). 74% (55/74) reported that the IGH Fellowship had an impact upon their career, 35 of which reported that the impact was “substantial”. The themes that emerged from the interviews revealed a personal development cycle that consolidated the fellows’ interests and values whilst enhancing their self-efficacy and subsequently impacted positively upon their career choices. Three interviewees expressed frustration at the lack of opportunity to utilise their new skills on returning to the United Kingdom (UK).

**Conclusions:**

The IGH Fellowship successfully empowered healthcare professionals to self-identify as leaders. Of the 45/74 respondents who commented on the impact of the IGH Fellowship on their career, 41/45 comments were positive. The fellows described a process of experiential learning, reflection and evolving cultural intelligence, which consolidated their interests and values. The resultant increase in self-efficacy empowered these returned fellows in their choice of career.

## Background

In the twenty-first century the NHS faces unprecedented challenges that differ from those it faced when it was first conceived in the 1940s [1, 2]. It is acknowledged that in order to be effective and responsive to these challenges there need to be people with established leadership skills embedded within the NHS at all levels [1, 2]. In his 2008 report, “High-quality care for all: NHS next stage review”, Lord Darzi emphasised the importance of fostering clinical leadership in order to create the right environment for high-quality care [1]. Due to the complex nature of leadership development, the challenge lies in integrating these skills effectively into NHS staff training and empowering leadership at a local level.

Leadership development needs to be encouraged and accessible for all members of the multidisciplinary healthcare team [1]. In recent years there have been a number of initiatives to increase leadership capabilities across the NHS [3], an example of which is the NHS Healthcare Leadership Model, developed in 2013. This model aims to empower healthcare professionals of all backgrounds in developing their leadership behaviours [4]. Until recently the integration of medical leadership and management (MLM) in undergraduate and postgraduate medical training was limited [3, 5]. With increasing awareness of the importance of MLM, and of embedding good leadership practice early in the careers of healthcare professionals [1], medical schools have begun to integrate this teaching into their curricula [5]. The effectiveness of different interventions in developing leadership skills has been explored, however there is limited high-level evidence supporting them [3]. Giving potential leaders challenging assignments, with mentor support, has been shown to be an effective way of developing leadership skills [6, 7]. In his paper on the future of leadership development, Nick Petrie describes how individuals develop faster when they are given responsibility for their own development and provides a strong argument for increasing emphasis on vertical development (which describes development earned for oneself) [8]. Assignment-based leadership development links with Kolb’s experiential learning process in which the individual moves from concrete experiences to reflection, abstract conceptualisation and, finally, active experimentation which is known to be a powerful form of adult learning [9].

The Improving Global Health (IGH) programme was developed with three key objectives: firstly, ‘to support the delivery of sustainable improvements in health and healthcare, in collaboration with overseas partners in their community in resource poor settings’, secondly, ‘to provide an unparalleled personal and leadership development experience for participants (fellows) who are recruited as volunteers on the programme’ and thirdly, ‘to create a cadre of leaders with service improvement skills who are able to make a real difference to the NHS on their return to the UK’ [10]. Central to the programme is the opportunity for fellows to lead a quality improvement project with an overseas partner thus learning leadership skills and behaviours. During this assignment fellows live in a resource-poor country for a period of three to nine months. The placement forms the fulcrum of the fellow’s experiential learning cycle as they act and reflect upon their leadership performance within the new environment. Throughout the programme fellows are supported by UK-based mentors who help them to identify their learning needs and support them through the development process. There is also a period of formal leadership training prior to overseas placement to optimise assignment-based learning. During this process the NHS Healthcare Leadership Model [4], and the models that preceded it, are used to define leadership and aid the Fellow in identifying and addressing their learning needs.

The purpose of this evaluation was to understand the impact of the IGH Fellowship on the leadership development of returned fellows and on their subsequent careers. In particular, we wanted to understand the process of personal development in order to further improve the aspects of the IGH Fellowship that facilitated leadership development.

## Methods

### Participants

All fellows who completed an IGH Fellowship between August 2008 and February 2015 were identified and invited to complete an online questionnaire regarding their experience.

### Questionnaire

The questionnaire was designed to elicit information regarding the individuals’ motivation for applying for a fellowship, the development of fellows’ leadership skills and the impact on the fellow’s careers after completion of the programme. In addition, it identified respondents’ demographic and professional background. The questionnaire used a mixture of structured and non-structured questions to collect data and explore the respondents’ ideas. Prior to dissemination, the survey was beta-tested by senior IGH Fellows and revised following recommendations. The survey was implemented online through SurveyMonkey**©** between the 17th August 2015 and the 5th October 2015.

Microsoft Excel® proprietary software was used to analyse respondents’ demographic and professional details. The non-structured free text responses were explored through an inductive approach utilising thematic content analysis. The text initially underwent open coding which was subsequently refined to develop a final coding framework for each of the key questions posed.

### Semi-structured interview

Fifteen respondents were identified through matrix selection and invited to undergo a semi-structured interview to explore the main themes that emerged from the questionnaire results. The interviews were conducted by one of two individuals, were tape recorded and transcribed. At the beginning of each interview the purpose of the evaluation was explained and confidentiality assured. After clarifying demographic and professional details, the interviews explored the general impact of participation in the programme, the skills acquired and the impact of the programme on the interviewee’s current and future career aspirations.

The interview transcripts underwent open coding that was refined into a final coding framework as seen in Table 1. Each transcript was coded independently by two researchers; in the event of conflict, a third researcher coded the response to resolve the disagreement. The transcripts were analysed within this framework and grouped together by theme. All transcripts and quotations were anonymised prior to analysis to ensure confidentiality.

**Table 1:** Final coding framework for content analysis by theme

Theme	Code	Description
Experience factors	Experience	Being in a position to practice leadership skills, facing challenges and having to adapt to new roles whilst leading an overseas quality improvement project.
	Exposure	Exposure to other cultures and health professionals
	Leadership styles	Exposure to and an awareness of different leadership styles
Personal development factors	Interests	Development and consolidation of interests, aspirations or values both personal and professional
	Perspective	Perspective on professional development and the NHS
	Insight	Insight into personal attributes and cultural norms and how these influence behaviour
Internal outcomes	Personal Qualities	Development of qualities such as confidence, innovation, flexibility, adaptability and resilience.
	Change in practice	Active changes to the fellow’s professional practice
	Change in career choice	Active changes in career direction or career ambitions
External outcomes	Perception	Perceptions of the IGH Fellow from others
	Opportunity	Relating to the availability or lack of availability of opportunities on return to the UK
	Impact of Fellowship	Relating to the impact of the IGH Fellowship on the fellow’s career

## Results

### Questionnaire

#### Respondents

The questionnaire was distributed to 111 returned IGH fellows and was fully completed by 74 (response rate: 67%). Sixty-four respondents (86%) were female, reflecting the gender split of those applying for the fellowship. The greatest number of respondents were aged between 31 and 35 years (31 respondents) followed by those aged between 26 to 30 years (25 respondents). This reflects the programme’s focus on healthcare professionals who are at the early stages of their careers. In order to participate in the fellowship 23 respondents (31%) took a career break, 25 (34%) planned a gap in their post-graduate training, eleven (15%) waited for the end of their contract, eleven (15%) successfully planned the IGH Fellowship as a secondment from their employment and four (5%) resigned from their jobs.

The opportunity for overseas experience and personal development were the most important motivations cited for applying for the IGH Fellowship (Table 2). Leadership development was the third most frequently cited motivation for application to the programme.

**Table 2:** The frequency of motivating factors cited for application to the IGH Fellowship

Motivating factors for application to the IGH Fellowship	Frequency of citation
Overseas experience	52 (70%)
Personal development	24 (32%)
Leadership development	18 (24%)
Quality improvement	16 (22%)
Looking for inspiration	13 (18%)
Public health	9 (12%)
Recommendation	5 (7%)
Management skills	3 (4%)

There were a range of professions represented in the group of respondents. The largest group were doctors working in secondary care (32/74) followed by doctors working in primary care (18/74). In addition, there were physiotherapists (6/74), professionals working in the public health sector (5/74), nurses (4/74), managers (2/74), midwives (2/74), occupational therapists (2/74) and a pharmacist (1/74), dietician (1/74) and podiatrist (1/74).

### Placements

Sixty-seven respondents (91%) completed one placement and seven respondents (9%) completed two, resulting in a total number of 81 placements. All placements were completed between 2008 and 2015. The majority of first placements were between four and six months in length (56/74), with 15% (11/74) undertaking placements of less than three months and 9% (7/74) undertaking placements lasting over six months. Thirty-five respondents (47%) undertook their first placement in Cambodia, 29 (39%) at one of three locations in South Africa, ten (14%) in Tanzania, two in Kenya and one in Zambia. These numbers may reflect the longevity and nature of the partnerships with organisations in these countries. The work undertaken by respondents while on the overseas placements spanned many themes, the most commonly reported were quality improvement (85%), public health (77%) and education (72%).

### Leadership development

**Fig. 1:**
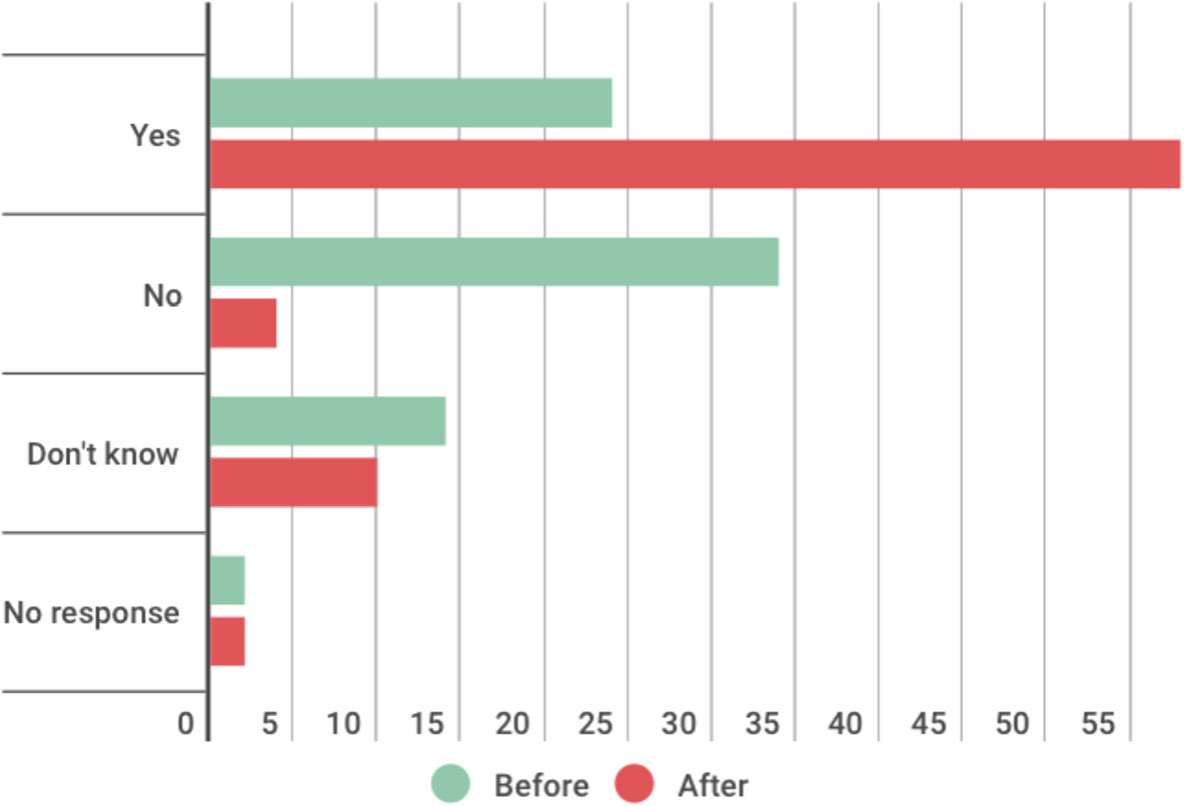
Self-perception as a leader before and after the IGH programme

Twenty-four respondents (32%) considered themselves leaders prior to their participation in the programme compared with 58 (78%) following it (Fig. 1). An analysis of the non-structured free text responses revealed that the most commonly cited reasons for the shift in self-perception was a change in the understanding of leadership and a development of personal qualities that empowered leadership. For example:

*‘I have seen how it is possible to lead from any role within an organisation, not just those traditionally seen to be “at the top”.’* (Respondent 39)

*‘I recognise the qualities of my personality that are positive when I am in a leadership role; I also recognise those aspects of my character that are a barrier to good leadership. Insight into both aspects has helped me going forward.’* (Respondent 66)

Ten respondents reported that they did not know whether they considered themselves to be leaders after completion of the programme, six of these had not considered themselves leaders prior to the programme. The majority of this group stated that their ability to act as a leader was context-dependent and a result of the opportunities that arose. For example:

*‘I think this depends on the situation – I have definitely further developed my leadership skills through the project but I think it is probably a little more complex than simply being a leader or not being a leader.’* (Respondent 47)

### Career impact

When asked whether they had been able to use the skills learned from their project work since completing the Fellowship, 53 respondents (72%) reported that they had been able to utilise these whereas thirteen respondents (18%) had not and six respondents did not know. Those who believed that they had not utilised these skills mostly cited that they had not yet had the opportunity to do so. One respondent expressed frustration:

*‘Partially I have been able to use skills learned, however my employer failed to see that I could do more if I had been given the right tools.’* (Respondent 17)

In contrast, those who had been able to use the skills that they had developed on the programme were very positive about the opportunities they had encountered.

*‘I have undertaken many different projects which have allowed me to use my leadership and service development knowledge.’* (Respondent 23)

Others described how these skills had enhanced their clinical practice:

*‘I think my self awareness of my leadership and management skills has greatly increased and I am much more effective as a doctor on the ward.’* (Respondent 33)

Fifty-five respondents (74%) reported that the IGH Fellowship had an impact on their career, with 35 of these defining the impact as “substantial”. Respondents who reported a substantial impact commonly stated that they had developed new qualities or had found broader career opportunities. For example:

*‘I would have probably left the NHS. [It has] given me confidence to view the world as my oyster and not hold myself back. [It has] opened the door to several contacts, opportunities and ideas.’* (Respondent 48)

For some it completely changed the direction of their careers:

*‘It has resulted in my completely changing my career direction. For a long time I had been unhappy with my job and had planned to leave the UK to work in Australia long-term. Since [the programme] I have been able to find a career that I find more fulfilling and I have become much more committed to remaining in the NHS long term.’*(Respondent 6)

Of the respondents who did not feel that the programme had an impact on their careers, some felt that it was too early to be able to assess the true impact of the programme, whereas others expressed frustration at the lack of opportunities available to them. For example:

*‘Whilst very much needed, I do not think that the NHS at the moment is fostering an appetite amongst its employees to embrace positive change and improvement.’* (Respondent 56)

### Semi-structured interview

#### Participants

Fifteen participants were selected from the questionnaire respondents. Thematic content analysis of the semi-structured interviews identified the process by which the fellowship influenced the fellow’s career and leadership development. In the qualitative section of this report, the term ‘most’ relates to ideas expressed by more than 75% of interviewees, ‘many’ relates to ideas expressed by more than 50% of interviewees and ‘some’ relates to ideas expressed by more than 25% interviewees. For analytical purposes, the themes were subdivided into concrete experience factors that instigated development, abstract personal development factors which resulted from personal reflection and the subsequent internal and external outcomes (as described in the Methodology Table 1).

### Experience factors that instigate development

Core to the IGH programme was the overseas placement in which the fellow had the opportunity to lead a quality improvement project under the direction of the in-country partner. Three aspects were identified by interviewees as being important to personal development: the experience of practicing leadership skills, facing challenges and adapting to new roles; the exposure to different professions, cultures and attitudes; and personal awareness of effective and ineffective leadership styles.

#### Experience

The benefits that interviewees described as a result of working on an overseas quality improvement project may be broadly grouped as those relating to the experience of working within a foreign healthcare system and those relating to the opportunity of practicing leadership through taking the lead on an assignment. Leaving the UK and facing the challenge of working within a foreign healthcare system gave the fellow a new perspective on healthcare:

*“…it makes you look at the wider picture as you turn up knowing nothing.”* (Interviewee 3)

Many of the interviewees reflected positively on the experience of working abroad. They described qualities that they developed as a result of being immersed in a foreign culture, such as flexibility, adaptation and self-reliance:

*‘…it made me more responsive to the environment that I’m in…’* (Interviewee 9)

*‘…you are quite self-reliant and you are leading this project so you have to make decisions and get people on board. That kind of attitude can be quite helpful.’* (Interviewee 6)

Most of the interviewees stated that that the opportunity to lead a quality improvement project allowed them to practice their leadership skills. It was commonly believed that such skills could not be taught in the classroom:

*‘It takes practice to [lead], it’s not something you can be taught.’* (Interviewee 10)

Interestingly, many of the interviewees stated that it allowed them to practice skills that would not have been possible for them in the UK.

*‘…you are sprung into a leadership role when you’re working on the programme and you’re doing things like training staff, capacity building and leading projects. These are things you would never have a chance to do at that level of seniority in the UK.’* (Interviewee 2)

All of the interviewees agreed that the experience was a valuable one and was positive in developing their leadership skills. The local expectations and the high level of supported autonomy contributed to the perceived effectiveness of the fellow’s development:

Interviewee 7:

*…you are suddenly the lead on a project, it is very much in your court in terms of how you go about that project. In [XXXX], people were looking to you to be the leader and to come up with the direction and strategy for it.* (Interviewee 7)

#### Exposure

The IGH programme exposed the fellows to other cultures, challenges and health professionals that the interviewees did not believe they would have experienced in their roles within the NHS. It was noted by the interviewees that were not working face-to-face with patients in the NHS that there was great value in working on projects relating to the practical delivery of healthcare. This allowed them to develop an understanding and appreciation of the challenges and demands from a different perspective:

*‘I was given the time and space to work through with the clinical team what they needed and how I could add value to the clinical pathways…’* (Interviewee 1)

Many of the interviewees cited that these interactions with professionals from different backgrounds influenced the way that they interacted with their colleagues on returning to work within the UK. The focus of these changes included an increase in empathy and willingness to understand another healthcare professional’s perspective, and an ability to adapt to other ways of working. For example:

*‘I’m probably more empathic since I’ve been back when people have had concerns and I was probably a bit more judgemental before I went…’* (Interviewee 3)

*‘The way I relate to others is different now…you were forced to rely on other people to share decisions and I’ve brought that function into my work at the moment…’* (Interviewee 15)

Most of the interviewees asserted the value of exposure in aiding them with their development and their careers:

*‘[The IGH programme] has definitely helped [my career]…Lots of things: I’ve been exposed to different types of people, styles of work, teams, yes, absolutely helped.*’ (Interviewee 6)

*‘...being exposed to people, that was definitely something very beneficial and that I wouldn’t have had without the Fellowship.’* (Interviewee 8)

### Leadership styles

Repeatedly cited by many of the interviewees as an important aspect of the fellowship was the fellow’s exposure to different styles of leadership. Most interviewees stated that they were able to observe different leadership styles and reflect upon their effectiveness. Leadership behaviours were observed not only in the overseas partners, but also in the UK-based IGH programme directors and the other IGH Fellows. Some interviewees discussed how they reflected on the leadership styles embraced by the individuals they encountered. The following comment does not explicitly state whether the leaders referred to are part of the overseas partnership or the UK-based programme:

‘*Our big boss had a very unusual, interesting leadership style. Our local boss had a very different leadership style. They both had strengths and weaknesses and I think observing and working with them you could see what worked in a very different context.’* (Interviewee 2)

Some of the strengths that were highlighted related to motivation and energy, communication and the ability to unify a team. One interviewee noted:

*‘These people I’m thinking of managed to unify people so nobody was upset or offended and got things back on track. It was great to see that that it is possible - how you can navigate situations and keep everyone happy whilst still moving things forward.’* (Interviewee 8)

Though the leadership styles were not always thought to be positive, many of the interviewees explained that the learning they gained was still valuable. For example:

*‘I could see different ways leaders went about it and there is something to be learnt both positive and negative’*. (Interviewee 7)

Many of the interviewees expressed how their reflections on seeing different styles of leadership influenced the way that they viewed their own leadership abilities and skills.

*‘I didn’t grasp the concept that it might be much more collaborative and that you could still be a leader but an integral part of the team I had my eyes opened to different styles of leadership…’* (Interviewee 5)

Through their reflections most of the fellows were able to consider the ways in which they could influence their effectiveness as leaders and develop their own style.

*‘I am more aware of the leadership styles of others and how that may impact on the desired results that they would like. Equally, I am much more aware of how I come across to others and reflecting on when things maybe haven’t gone so well.’* (Interviewee 3)

### Personal development factors

As a result of the experience factors described above, the interviewees described areas in which they had undergone significant personal development. Over the course of our interviews, three of these areas were identified: interests, relating to the evolving interests and values of the fellow; perspective, relating to their professional development and their role within the NHS, and insight, reflecting the fellow’s self-awareness.

### Interests

Interviewees described how they developed their interests and aspirations as a result of their experiences on placement. Common areas that evolved included interest in leadership roles, developing the service, improving patient care and global health. Interviewees reported that the development of these interests and values influenced their subsequent choices in careers. One interviewee stated how the programme equipped her to pursue the career of her choice:

*‘The programme taught me how to project myself, how to run a project and gave me an idea or concept of how to do this and it has made me more able to carve my career niche… which is very useful for me.’* (Interviewee 12)

Many interviewees reported that they had established new ideas about roles that they would like to work in and empowered them to pursue their career choices. For many, it enabled them to understand how they would be able to achieve such a goal:

*‘It gives you more understanding of yourself and lets you figure out what you can do.’* (Interviewee 12)

*‘I would consider roles that I wouldn’t have considered if I hadn’t done [the IGH Fellowship].’* (Interviewee 6)

### Perspective

The opportunity to work on a placement outside of the NHS allowed many of the interviewees to gain valuable perspective on both their careers and the way that their local NHS system functioned when they returned. Reflecting on how they felt prior to commencing their fellowship some of the interviewees expressed frustration at working within the NHS:

*‘It’s very easy when you’re working in such a big system to feel powerless, as if you can’t change anything…’* (Interviewee 2)

*‘An NHS hospital can be quite a difficult place, things don’t work the way you want them to and you can get frustrated and want to change everything, and, before I went away I got really frustrated by all these things, wanting things to be better and things to work better.’* (Interviewee 12)

There were both positive and negative attitudes towards the NHS expressed by the interviewees following the fellowship. Many explained that the experience of working in a foreign system allowed them to gain an appreciation for working within the NHS:

*‘We are really lucky in the system we have. Yes, there are lots of frustrations in the NHS but when you’ve seen other healthcare systems that are so basic and so different it makes you very grateful.’* (Interviewee 7)

*‘We have nice structured clinical times. We have access to everything we would need. Our patients have good levels of comprehension. We’re well paid for what we do…’* (Interviewee 3)

The other areas that interviewees found a new appreciation for included the evidence-based approach to medicine and the protocols designed to standardise patient care. On returning to the NHS, some of the interviewees explained how this new perspective empowered them to seek opportunities to help develop the service:

*‘When you work with IGH you realise how easy it is to get involved with things and change things and work at quite a high level, it gives you confidence to do that…’* (Interviewee 2)

*'I am much more knowledgeable now who needs to endorse changes and, if I see something not working very well, who I need to speak to for change. Now, I have the confidence to move higher up the chain to speak to people if I think something could be done better. Now, I’m more likely to say let’s not just talk amongst ourselves, let’s go out there and raise it higher up and get something done.’* (Interviewee 11)

Conversely, some of the interviewees took a more negative view of their new perspective on working in the UK. The majority of these views were due to frustration. For example:

*‘When I first came back and people were complaining, I was quite irritable and thought, “you don’t have any idea, you should just get on with it”…I got really angry once…’* (Interviewee 3)

Many of the interviewees explained how their new perspective allowed them greater scope in considering the challenges of the NHS and aided them with their approach to dealing with these challenges.

*‘I am able to look at the bigger picture much more…*’ (Interviewee 7)

*‘I definitely have better appreciation of the fact that there is more than one way to do things, just because somebody disagrees with me doesn’t mean I’m wrong and just because I disagree with someone it doesn’t mean they’re wrong…’* (Interviewee 15)

*‘It’s just stepping outside and seeing that things can function perfectly well using different models, that’s been a valuable experience’* (Interviewee 15)

### Insight

In addition to gaining perspective on their careers and the NHS, many interviewees believed that they had developed personal insight through reflective practice whilst completing the fellowship. In some instances this was expressed as a new awareness of qualities that they already possessed, and in other instances it involved recognising a gap between their perception of self and reality. Some interviewees reported that the programme empowered them to gain confidence in skills they had not previously recognised:

*‘I don’t think I viewed myself as a leader before I went away so I think it has changed even seeing myself as a leader, my ability to lead projects or come up with ideas... it gives you more understanding of yourself.’* (Interviewee 12)

For others, the insight they developed allowed them to focus on certain areas of their development:

*‘Before the programme I think I misjudged significantly how good I was or how bad I was at leadership skills in certain areas and I was surprised about this…[it] made me realise actually I do need to develop in some areas and I was wrong in my perception of where I was…’* (Interviewee 14)

Similarly, some interviewees reported that the improved self-perception allowed them to have greater insight into how they acted and were perceived by others. For example:

*‘I present and manage myself better… overall I think I’m much better at working with others and within teams than I was before…’* (Interviewee 11)

### Internal and external outcomes

As a result of the fellow’s personal development through the programme the interviewees identified various outcomes. These may be considered internal, in that they have influenced the behaviour of the fellow, or external, in that they influenced the perception of the fellow from outside sources. Internal outcomes included the development of personal qualities; change in practice and change in career choice. External outcomes included the perception of the fellow, the availability of opportunities and the overall impact of the fellowship.

### Personal qualities

The interviewees all reported on the qualities that they brought back with them to their work in the UK as a result of the IGH programme. Most commonly mentioned by the interviewees was confidence:

*‘I’m more confident, happier to direct people, listen to their feedback and give them feedback…’* (Interviewee 10)

Many related this new confidence with the ability to effectively work as a leader:

*‘I’m much more confident with leaderships-style tasks.’* (Interviewee 3)

Others directly related the confidence that they had gained to their choice to pursue jobs that they would not have applied for prior to the fellowship. One interviewee explained that the confidence she had gained had made her more articulate and enhanced her abilities to deal with conflict:

*‘I’m not scared to be fair at the risk of upsetting somebody, which I think has changed.’* (Interviewee 3)

In addition to confidence, other qualities that interviewees developed included a positive change to a more proactive attitude:

*‘I am more likely to think “what can I do to change that?” rather than “oh that was someone else’s fault” or “it couldn’t have been helped”…’* (Interviewee 6)

Similarly, others described the positive effects resulting from their ability to take the initiative:

*‘I’m far more likely to approach someone though before I may have lacked the confidence to do that…’* (Interviewee 5)

Adaptability and flexibility were additional qualities that were highlighted by the interviewees as new ways that they had learned to change their approach:

*‘I am more likely to change my behaviour, maybe to behave in ways I wouldn’t normally want to in order to try to work successfully with someone…’* (Interviewee 14)

### Change in practice

During each interview, the interviewees explored the ways that their development had influenced their subsequent approach to professional practice. This included practical changes, such as a fellow in a managerial position deciding to spend sessions working clinically in order to develop connections with the staff involved in practically delivering healthcare, and abstract changes in which the fellow adapted the way that they interacted with their colleagues.

One interviewee explained how choosing to spend some time working with clinicians had enhanced her practice as a manager in the NHS:

*‘I think that they have a lot more respect for me as I feel they think I’ve taken the time to understand their role and frustrations… my personal experience working so closely with clinicians has made me a much stronger advocate…’*(Interviewee 1)

Most commonly cited as a change in practice was the change to the interviewees’ interactions with colleagues in a team.

*‘Overall, I think I’m much better at working with others and within teams than I was before…’* (Interviewee 11)

When discussing changes in their approach to leadership, opinion was divided regarding how their behaviour had evolved. Most cited that their behaviours had changed considerably:

*‘It has proved a really good experience for knowing how to work with others in a team, how to psyche yourself to work with others, the importance of working as a team, sitting down, thrashing out ideas, taking other people point of view and bringing those on board to reach objectives…*’ (Interviewee 11)

Whereas others reported that they did not believe that their leadership behaviour had changed although they were able to reflect upon leadership styles:

*‘I don’t think it necessarily changed how I behaved as a leader but it definitely gave me a chance to reflect on the leadership styles I knew.’* (Interviewee 9)

### Change in career choice

Many interviewees described the differences between their career path prior to the fellowship compared to their career path following it, with 5/15 interviewees reporting a complete change in career direction. When discussing careers within the NHS, it was generally expressed that the structure for career progression was rigid and prescriptive. Whilst reflecting on this, one interviewee explained:

*‘I would have thought I was the kind of person who would have stayed on the treadmill and gone through quite quickly. So no, I never thought I would be doing what I’m doing now…’* (Interviewee 2)

Many interviewees revealed that their values had been clarified through their experience in regards to the type of organisation that they wanted to work for and in regards to the work-life balance that they would like to achieve. For example:

*‘I probably would have been more focussed on the job role, the banding and the status of that role, whereas now I am more bothered by the value set in the organisation, what its trying to achieve…’* (Interviewee 1)

Many of the interviewees explained that their career choices had changed direction following the fellowship. For some this meant applying for roles that were more senior than they would have prior to the fellowship, for some it involved applying for additional roles with a leadership or management aspect and for some it involved completely changing the course of their career. Many of them described it as a useful talking point for interviews and for their CV that set them apart from their peers:

*‘…so few people have had this experience, particularly in a management career in the NHS and quite early on in my career, it was part of my unique selling point.’* (Interviewee 1)

The experience allowed them to have solid evidence as a foundation for their credibility. For example:

*‘…if people do question it, I feel I am able to give them some examples of quite significant leadership I’ve done that most GPs haven’t had the opportunity to do at all…’* (Interviewee 7)

The majority reported that this was positive for their careers, however one interviewee explained:

*‘My midwifery career it probably hasn’t helped as I feel ready to move on from that.’* (Interviewee 8)

This reflects the frustration that was expressed by some of the interviewees in regards to the rigid and traditional clinical roles within the NHS. Some interviewees expressed new aspirations for their careers:

*‘…I purposely chose my role in xxxx Trust to be more strategic. It was a conscious effort having enjoyed the opportunity to step back from direct service provision; this was something I really valued and looked for in my current role.*’ (Interviewee 1)

*‘I can’t think of just going back to doing ward work without wanting to do something more exciting and challenging.’* (Interviewee 8)

When asked whether they intended to remain working within the NHS the majority of the interviewees were very positive that they would remain. Most reported a great affinity for the NHS despite the frustrations and difficulties they associated with working in it:

*‘I think the positives to our careers outweigh the negatives despite everything that’s going on.’* (Interviewee 11)

There were a few interviewees who were more pragmatic in their responses and highlighted a new awareness of their own transferable skills and the opportunities globally for healthcare:

*‘I have a real love for the NHS… and the opportunity to stay within the NHS is something I’d definitely welcome, but I am open minded enough to know that health is becoming more global.’* (Interviewee 9)

*‘I wouldn’t be afraid to leave medicine or hospitals and work for the NHS in terms of public health or work for another organisation. I do think that having had a high quality education as doctors, we are able to apply our skills to different areas and we do have skills that can be offered, not only in clinical work, and knowing that broadens your horizons a bit.’* (Interviewee 15)

### Perception

Through the course of the study, interviewees explored the ways that perceptions about them had changed as a result of the fellowship. There were mixed reactions reported with some colleagues expressing scepticism about the value of the fellowship and others expressing interest. Discussing these reactions, one interviewee explained:

*‘…people who value the work, the fellowship and the concept of what I did in [XXXX] value it and explicitly are very energised, encouraged and want to know more about it…*’(Interviewee 1)

Others had positive reactions from their colleagues:

*‘…the various [GP partners] recognise what I’ve done and have said this is the type of experience that stands and it’s what they look for more and more in potential new partners.’*(Interviewee 11)

*‘…they very much say I’ve changed – all for the better.’*(Interviewee 3)

Most of the interviewees said that their colleagues’ perception of them had changed following the fellowship, with some reporting positively the results of greater confidence, taking the initiative and being proactive. However, some were unsure whether perceptions about them had changed as they had not been established within a role for a long enough period of time to have received feedback.

### Opportunity

On returning to their careers in the UK, some of the interviewees reported that the fellowship had given them a unique platform from which further opportunities had developed. As one interviewee explained:

*‘…[the IGH Fellowship] enabled me to build a very great CV, very good networks and contacts and I don’t think I would have been able to have got to where I have without having some of the IGH education…’*(Interviewee 2)

For many it developed their ideas regarding their career choices and provided opportunities for the fellows to explore.

*‘It led on to other things and opened my eyes to other opportunities…*’(Interviewee 7)

In contrast, others expressed frustration at the lack of opportunities to utilise their skills or develop further in their NHS roles. When talking about opportunities for further development through performing audits and quality improvement in her clinical work, one interviewee explained:

*‘…it was a shame there wasn’t more use made of me. It’s a bit of a waste when you have someone come back who is very motivated to do the job which others aren’t particularly eager to do…’*(Interviewee 8)

Similarly, other interviewees expressed their frustration:

*‘…there is more I’d like to do but I’ve found it difficult to have the opportunity.’* (Interviewee 3)

*‘…I became increasingly frustrated as I didn’t see any opportunities in my role as a locum GP or in the wider NHS workforce…’* (Interviewee 7)

This frustration prompted Interviewee 7 to leave their current posts and seek personal development opportunities elsewhere in order to find a fulfilling role:

*‘…when I came back and got the job working for the CCG (Clinical Commissioning Group) I felt then that I did have opportunities for leadership back in the UK.’* (Interviewee 7)

Most of the interviewees expressed a desire to continue to develop and explore challenges that arose, with many of them actively seeking new opportunities.

### Impact of fellowship

When asked about the impact of completing the fellowship on their career, the majority reported a positive impact. Some interviewees highlighted the qualities that they had developed on the programme:

*‘…I would definitely use those qualities and use those skills I learnt in the future for leadership and clinical leadership.’* (Interviewee 6)

Others discussed the motivation and enthusiasm they gained from the programme:

*‘…I came back refreshed and renewed, motivated; all very positive type feelings to change. Yes, I think it was a great thing to do.’* (Interviewee 3)

Some of the interviewees explained that the skills and attitudes that they had developed through the programme would be increasingly important as they progressed through their careers, discussing their roles as consultants, GP partners and members of the CCG. One of the interviewees explained that it was too soon to understand the true impact of the programme on their career, the majority expressed that it had been very helpful:

*‘It’s immensely helped. There is no way I’d be where I am without it, its been absolutely crucial.*’ (Interviewee 2)

## Discussion

For this group of respondents successfully completing an IGH Fellowship empowered them to view themselves as leaders, developing and embedding qualities, behaviours and skills that not only enhanced their leadership abilities in their current roles but also encouraged them to pursue fulfilling career paths. In this discussion we ask why the programme was successful and try to delineate which elements encouraged the development of leadership skills and career choice. Answering these questions was essential to understanding how to develop leadership in healthcare for the future so that the IGH programme could optimise its effectiveness.

It is important to note that the individuals who undertook the IGH Fellowship self-selected through a desire to apply for the fellowship. Whilst leadership development was not the most frequently stated motivation for applying to the programme it was an important factor and so it is likely that the individuals undertaking the IGH Fellowship had some interest in leadership prior to commencing the programme.

The experience of completing an overseas quality improvement project was an important factor in enabling the fellow’s personal development. The cycle of experiential learning, through the process of experience, reflection, conceptualisation and experimentation was at the heart of the fellowship [9]. Creating an environment for experiential learning, through which knowledge may be created from reflection on experience, is known to be a successful form of adult learning [11]. In order for learning from experience to be effective, there needs to be a period of active reflection [12]. The opportunity for reflection was embedded in the structure of the IGH programme and was aided by individual mentors who supported the fellows in identifying their learning needs and developing strategies to address them.

The uniqueness of the experience provided by the fellowship was made up of two elements: its overseas nature, involving significant cross-cultural work within a foreign healthcare system, and the opportunity to lead a quality improvement project at an early stage of the individual’s career. The capability to work effectively across cultures is dependent upon an individual’s cultural intelligence [13]. This relates to the ability to adapt to the nuances of different cultures and consists of three core elements: metacognition and cognition (described as thinking, learning and strategizing), motivation (described as efficacy and confidence, persistence, value congruence and affect for the new culture) and behaviour (described as social mimicry and behavioural repertoire) [14]. In order to be successful in their overseas placements whilst working within a foreign culture, the IGH Fellows would have been required to utilise and enhance their cultural intelligence. It has been postulated that employing cultural intelligence enhances the likelihood that individuals will engage in experiential learning and this will then guide the direction of their development [15]. As such, the integrated nature of the overseas placement is likely to have provided a fertile environment for significant personal development.

The second aspect of the overseas placement that provided a unique experience for the fellow was the high degree of responsibility for a project that they would not have had the opportunity to lead on whilst in their role prior to the fellowship. In order to be successful in a new environment and whilst undertaking a new task requires an individual to be highly adaptable, versatile and tolerant of uncertainty [16]. This adaptability is being recognised as an increasingly important trait for new leaders [16, 17]. The interviews reflect the process by which the fellows recognised the requirement to adapt in order to perform successfully on placement. As a result of this they enhanced their self-awareness, recognised gaps in their capabilities and developed qualities that enhanced their self-efficacy on returning to the UK. By taking an active role in their own development, the fellows were able to own their progress and success [8].

The majority of the fellows (58/74) who undertook the IGH Fellowship viewed themselves as leaders after completing the programme. In part, this was due to a new awareness of what they believed being a leader entailed, often moving away from a top-down, ‘heroic’ form of leadership to a more integrated, ‘shared’ leadership. The fellows were also able to undertake practical experience in leadership, which has been shown to be highly valuable in developing leadership skills [3]. In addition to developing an active awareness of leadership as a concept, and practicing their own leadership skills, the fellows were able to actively observe different leadership styles. This type of observational learning is also known to be influential in developing concepts of leadership [18].

The process of experiential learning empowered the fellows to explore and establish their interests, gain a valuable perspective on the NHS and develop their self-awareness. These factors allowed the fellows to cultivate personal qualities in relation to leadership and subsequently adapt their professional practice. In addition, it encouraged them to establish what they required from a fulfilling career. Following the fellowship many of the fellows reported that their career choice had changed. This was often regarded as a result of a change in their perception of self-efficacy, referring to an individual’s belief in their capabilities to perform a task. Self-efficacy is a dynamic set of self-beliefs with complex interactions that, in combination with outcome expectations (anticipating the consequences of particular actions and behaviours) and the formation of interests, influence an individual’s choices of activity [19, 20]. Self-efficacy is dynamic, and is influenced by personal performance accomplishments, vicarious learning, social persuasion and physiological states [19]. Personal accomplishments are believed to be the most influential of these and so personal success experiences will raise efficacy [19]. This may explain the increase in confidence reported by the majority of the interviewees following completion of the programme and their corresponding increase in perceived self-efficacy in relation to leadership roles and therefore their subsequent career choices.

The fellows who found or sought out new opportunities within their careers on returning to the NHS reported success and satisfaction. In both the questionnaire and the interviews, those who reported a lack of opportunity to utilise their new skills expressed frustration. This is an important point in terms of retaining staff within the NHS. Studies have shown that creating an employability culture, through stimulating employees, encouraging self-development and providing challenging work assignments, encourages staff retention [21]. In addition, for an organisation such as the NHS, which increasingly requires adaptability, change and leadership at a local level, it is essential to create opportunities to develop these skills for staff [1]. The results highlight that despite widespread agreement in the literature that NHS staff should be supported and encouraged to embrace leadership roles within their organisations [1–3], there remain some barriers to this at a local level. This poses the risk that empowered individuals who are keen to utilise their new skills will seek out fulfilling job roles outside of the NHS. It is interesting to note that despite developing their confidence in leadership, the individuals who expressed frustration at the lack of opportunities on returning to the NHS had not managed to successfully develop these opportunities themselves.

A systematic review of leadership development programmes for physicians in 2014 concluded that the majority of these programmes resulted in an increase in self-reported knowledge and expertise, as has been revealed in our study [22]. There are two aspects of the IGH Fellowship which contrast with the programmes evaluated by Frich et al. Firstly, the IGH Fellowship purposefully integrated the development of non-physician and physician professionals in the programme, which was found to have important developmental impact through shared experiences. Secondly, the primary learning method employed by the IGH Fellowship was through participation and experience rather than more common methods, such as lectures, seminars and group work. Quantitatively and qualitatively evaluating the impact of the IGH Fellowship provides an example of a novel programme which addresses some of the important gaps identified by Frich et al.

This evaluation has some limitations: 1) despite having a good response rate to the online questionnaire (67%) the absolute number of questionnaire respondents is small due to a limited overall number of fellows, 2) a correspondingly small number of in-depth interviews were conducted, though this made up 20% of all questionnaire respondents and accurately reflected the diversity of the individuals undertaking the fellowship. 3) As the purpose of the questionnaire and the interview were both explained in order to inform consent to participate, this may have introduced responder bias in which the respondents’ answers were influenced by this knowledge. 4) Though there are few similarly-structured programmes (i.e. focussing on an overseas placement as the catalyst for leadership development) with which to triangulate our findings, this study adds to the literature available for the development of leadership within healthcare [22] and to the development of leadership within the realm of global health. It is important to note that despite completing a programme with a strong global health aspect, the respondents and interviewees did not report pursuing further opportunities in global health; it is unclear whether this was due to desire for such opportunities or the lack of opportunities.

This formal evaluation of the leadership development aspect of the IGH Programme will enable the programme team to better understand the programme so that it can be further improved.

## Conclusions

The IGH Fellowship successfully fosters healthcare professionals to recognise and develop their leadership skills and behaviours and subjectively has a positive impact on their career development. The process involves experiential learning, reflection and evolving cultural intelligence, which in turn helps to develop self-awareness, increases self-efficacy and subsequently leads to positive changes in career choice. Three interviewees reported that they felt that their skills were not recognised on returning to the UK, suggesting a disparity in the perceived opportunities to utilise these skills on returning to the NHS. In order for the NHS to face the significant challenges of the twenty-first century, it is essential that healthcare professionals are supported in taking on leadership roles in the early stages of their careers. For the 41/74 fellows who self-reported positive career impacts through finding or creating opportunities to utilise the skills that they developed on the programme, valuable outcomes in terms of engaging with quality improvement work and leadership have been reported. It is essential that continuing evaluation is conducted into the effectiveness of leadership development programmes and the perceptions of such programmes to pave the way for innovative leadership within the NHS, whose survival is dependent upon the strengths of its staff and its ability to adapt to the changing environment.

## Abbreviations

CCG: Clinical Commissioning Group
CV: Curriculum vitae
IGH: Improving Global Health
MLM: Medical Leadership and Management
NHS: National Health Service
UK: United Kingdom
